# Supplementary material for: Low-temperature grapho-epitaxial La-substituted BiFeO3 on metallic perovskite
Source: Nat Commun. 2024 Jan 11;15:479. doi: 10.1038/s41467-024-44728-y (PMC10784590; doi:10.1038/s41467-024-44728-y)
Supplement: Supplementary file 1 — Supplementary Information [file 41467_2024_44728_MOESM1_ESM.pdf]

# Supplementary information

# Low-temperature grapho-epitaxial La-substituted $BiFeO_3$ on metallic perovskite

Sajid Husain[1,7], Isaac Harris[2,7], Guanhui Gao[3], Xinyan Li[3], Peter Meisenheimer[4], Chuqiao Shi[3], Pravin Kavle[1,4], Chi Hun Choi[3], Tae Yeon Kim[4], Deokyoung Kang[4], Piush Behera[1,4], Didier Perrodin[1], Hua Guo[3], James Tour[5], Yimo Han[3], Lane W. Martin[1,4,8], Zhi Yao[6], Ramamoorthy Ramesh[1,2,4,8]

[1]Materials Sciences Division, Lawrence Berkeley National Laboratory, CA 94720, USA.

[2]Department of Physics, University of California, Berkeley, CA 94720, USA.

[3]Materials Science and NanoEngineering, Rice University, Houston, Texas 77005, USA.

[4]Department of Materials Science and Engineering, University of California, Berkeley, CA 94720, USA

[5]Department of Chemistry, Rice University, Houston, Texas 77005, USA.

[6]Applied Mathematics and Computational Research Division, Lawrence Berkeley National Laboratory, CA 94720, USA.

[7]These authors contributed equally.

[8]Present address: Materials Science and NanoEngineering, Rice University, Houston, Texas 77005, USA.

Emails: rr73@rice.edu, jackie_zhiyao@lbl.gov, shusain@lbl.gov

Supplementary Note 1. X-ray diffraction of BPBO/LBFO heterostructures................................................ 2

Supplementary Note 2. Work function and band diagram in BPBO and SRO-based heterostructures. .... 11

Supplementary Figure 1-14

## Supplementary Note 1. X-ray diffraction of BPBO/LBFO heterostructures

X-ray diffraction (XRD) spectra of growth temperature dependent BPBO (50nm)/LBFO (40nm) samples shown in Supplementary Fig. 1a, where the deterioration of BPBO is observed above 500°C ((001) peaks is fully disappeared). Then the temperature was fixed to 450°C and oxygen partial pressure dependent BPBO (40nm)/LBFO (100nm) series was deposited (XRD Supplementary Fig. 1b, c). The lattice parameter values with respect to oxygen pressure shown in Supplementary Fig. 1d, and the best crystallographic quality sample was found to be at 50mTorr with smaller lattice constant than the bulk BFO[1], which is expected due to the La doping[2], however slightly larger than the SRO/BLFO high temperature sample (main text). The charge leakage was tested in a few representative samples as shown in current vs voltage curve (Supplementary Fig. 1e) and as expected, the lowest leakage current is found to be in the sample deposited at 50 mTorr.

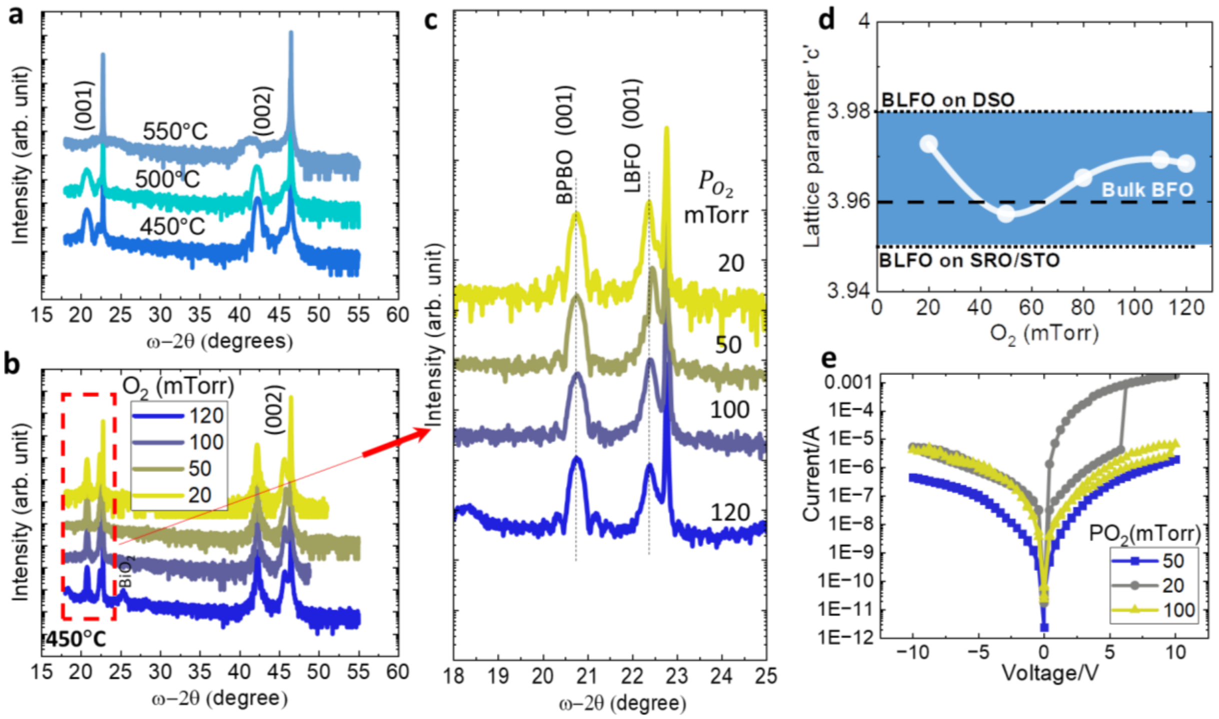


**Supplementary Figure 1|** X-ray diffraction patterns of BPBO/BLFO grown **a** at different temperatures and **b** at different $O_2$ pressures. **d** Lattice constant calculated using the (002) BLFO peak from **c**. **e** Current-voltage curves measured in the sample prepared under different oxygen partial pressures.

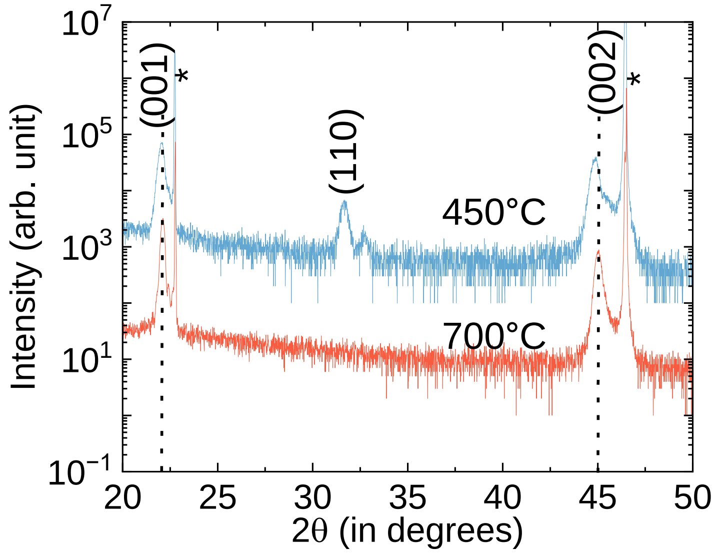


**Supplementary Figure 2|** X-ray diffraction patterns of BFLO thin film deposited at 450°C and 700°C temperatures on STO (001) substrate. Epitaxial growth preferred at high temperature on STO substrate whereas the additional phase appeared at low temperature. Peak broadening and slightly redshift in the (002) peak of 450°C BLFO implies the significant lattice deformation at low temperature. * Represent the substrate peaks.

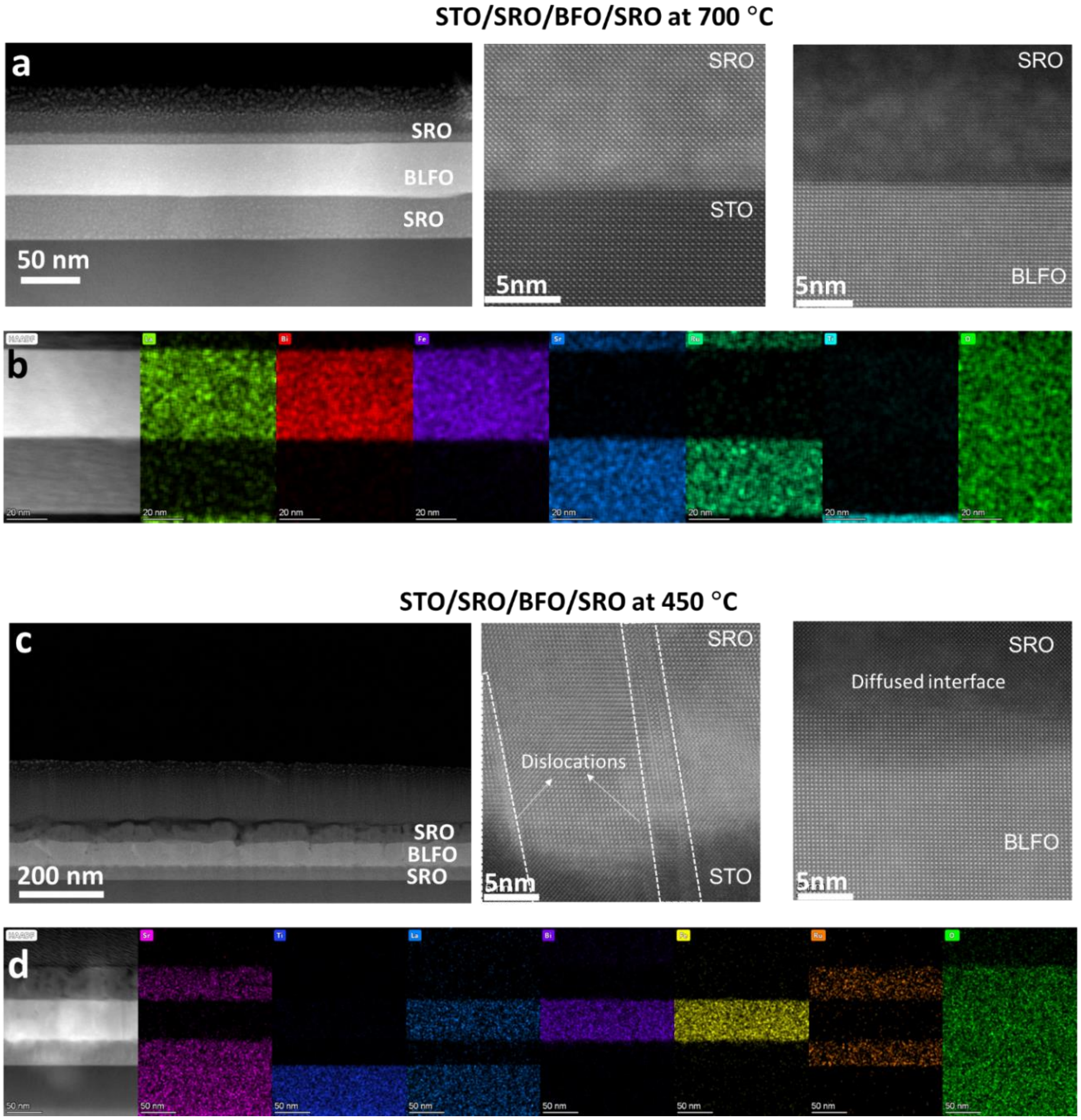


**Supplementary Figure 3|** HR-TEM images recorded on the samples **a** STO/SRO/BLFO/SRO and **c** STO/SRO/BLFO/SRO deposited at 700°C and 450°C, respectively. **b**, **d** Electron dispersive x-ray spectroscopy images are shown at the bottom panels.

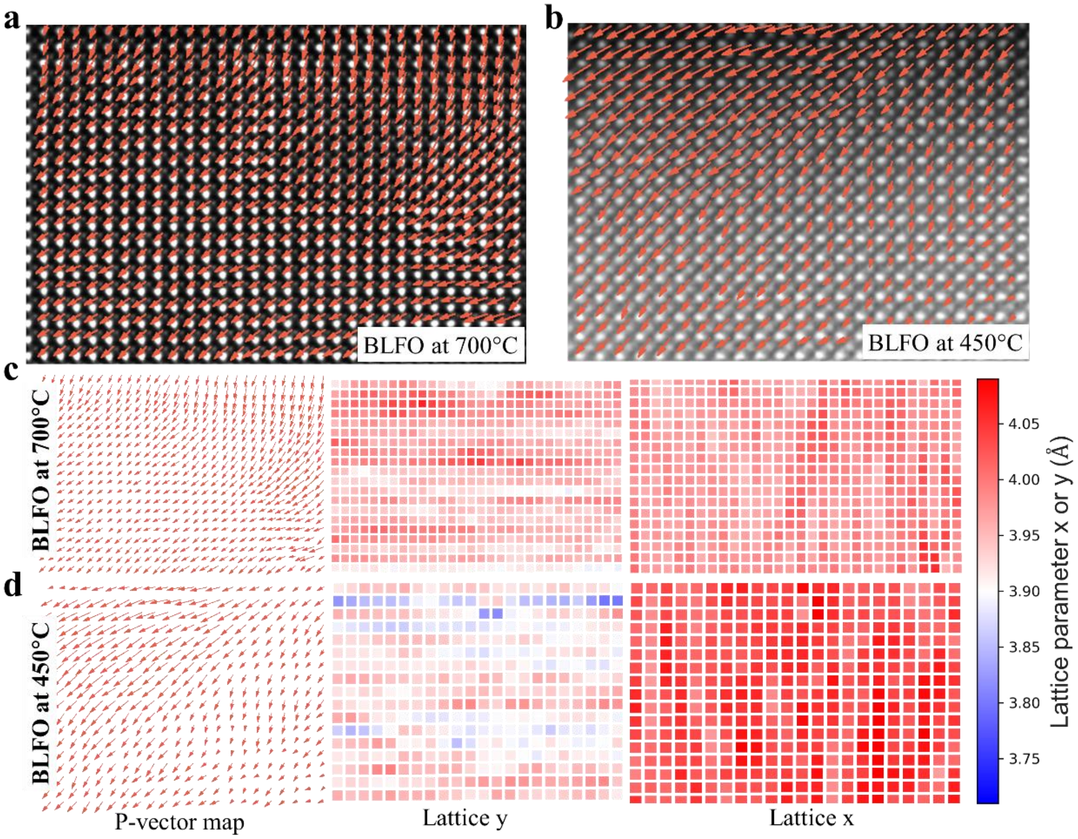


**Supplementary Figure 4|** HAADF-STEM imaging and polarization vector mapping on BLFO deposited at **a** 700°C on SRO, and **b** 450°C on BPBO. **c**, **d** The polarization vector map (on left) and the lattice parameters along x and y.

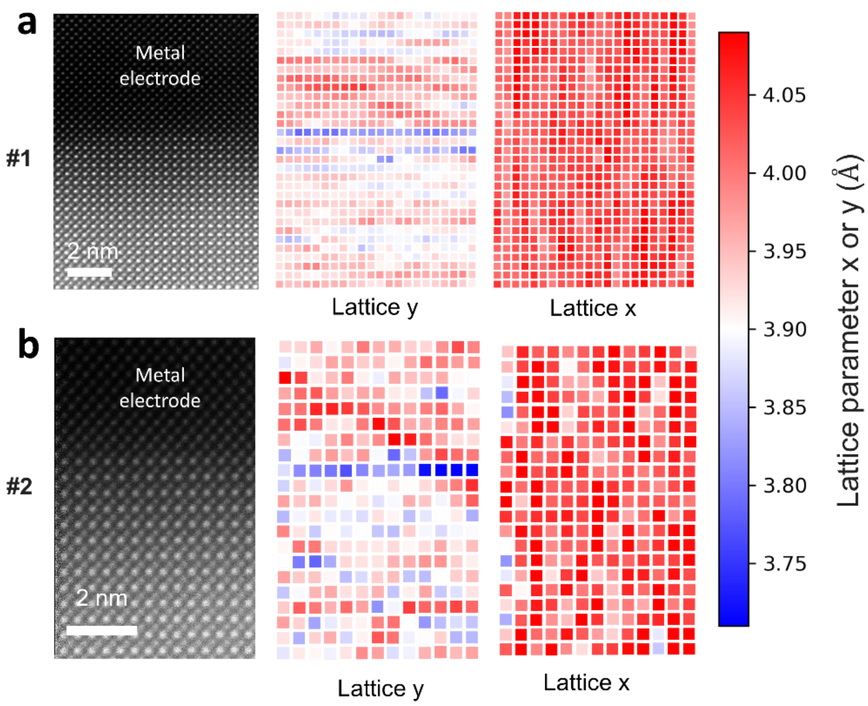


**Supplementary Figure 5|** Lattice parameter along x and y at different positions (marked as #1 '**a**' and #2 '**b**') in BFO deposited at 450°C. It depicts the short-range ordering of the polarization in low temperature deposited thin films.

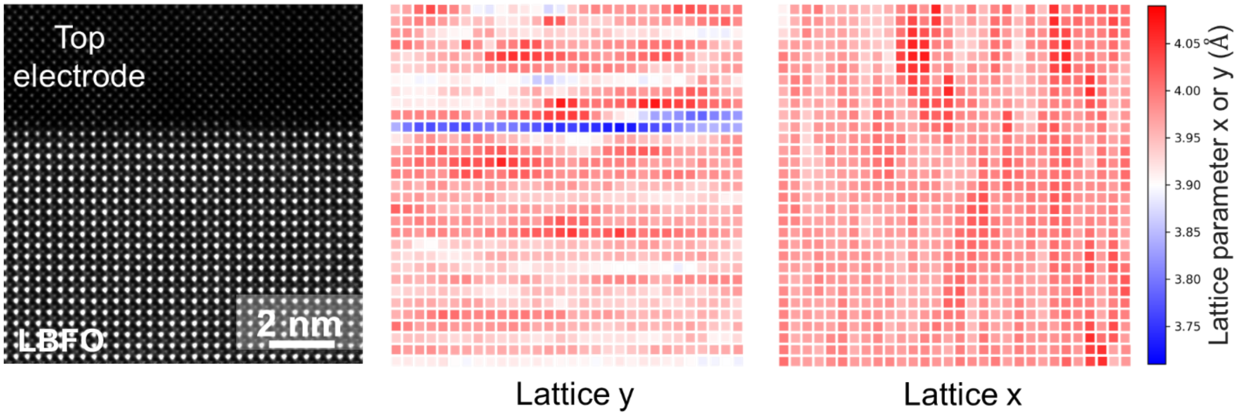


**Supplementary Figure 6|** Polarization displacement vector mapping and its uniformity in projection along x-and y in the lattice indicates the long-range ordering of the polarization in high temperature BLFO. The polarization mapping at the interface shows the finite deviation of the displacement vector which can be ruled out due to finite variation at one unit cell interface (one-unit cell of top BLFO surface and bottom surface of metal layer).

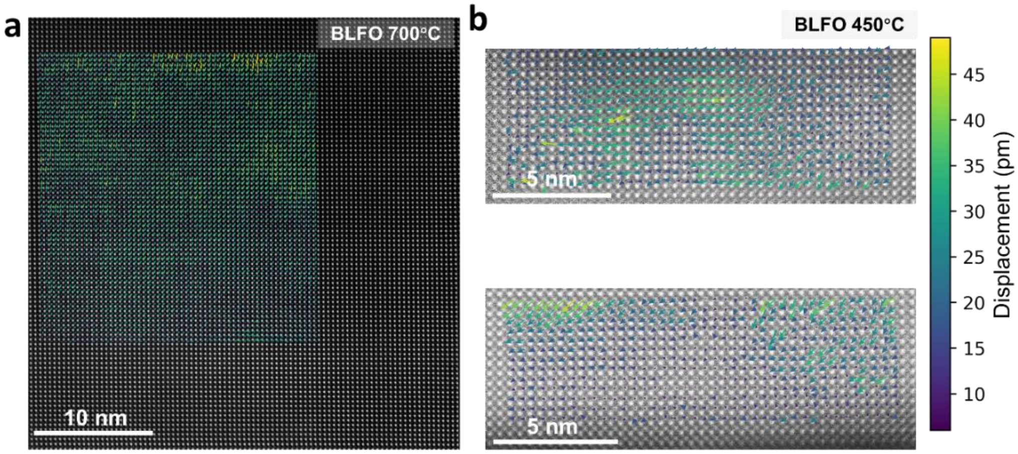


**Supplementary Figure 7|** Atomic imaging of samples deposited at **a,** high (on SRO) and **b**, low (on BPBO) temperatures. The colored arrows represent the polarization vector map. The mapping window is limited (in the area, left) due to the defocusing issue in the big cross-section. It might have occurred due to the ion milling damage and is more pronounced in the low temperature deposited BLFO (on BPBO).

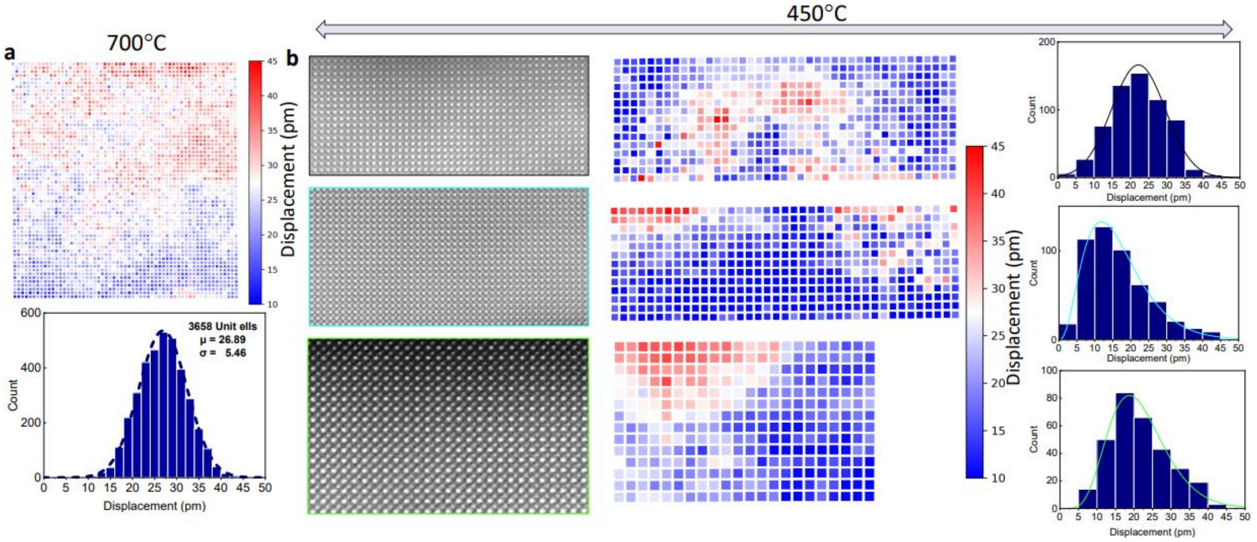


**Supplementary Figure 8|** Polarization vector map distribution in high and low temperature BLFO represents the long- and short-range ordering. One colored square in the map corresponds to a single unit cell. The displacement vector distribution is reproduced from the main text Fig.2. In the big area map, **a**, BLFO shows the long-range polarization ordering, whereas the selected area mapping in the 450°C BLFO sample in **b** (from Fig.2e), the polarization color map has scattered view and do not show the continuous change (in small cross-sections) of the polarization. This depicts the short-range polarization ordering, which is likely due to the relaxed epitaxy. 1 square corresponds to 1 unit cell of BLFO. The polarization displacement distribution plots for high and low temperature represent the short-range and long-range ordering in the BLFO.

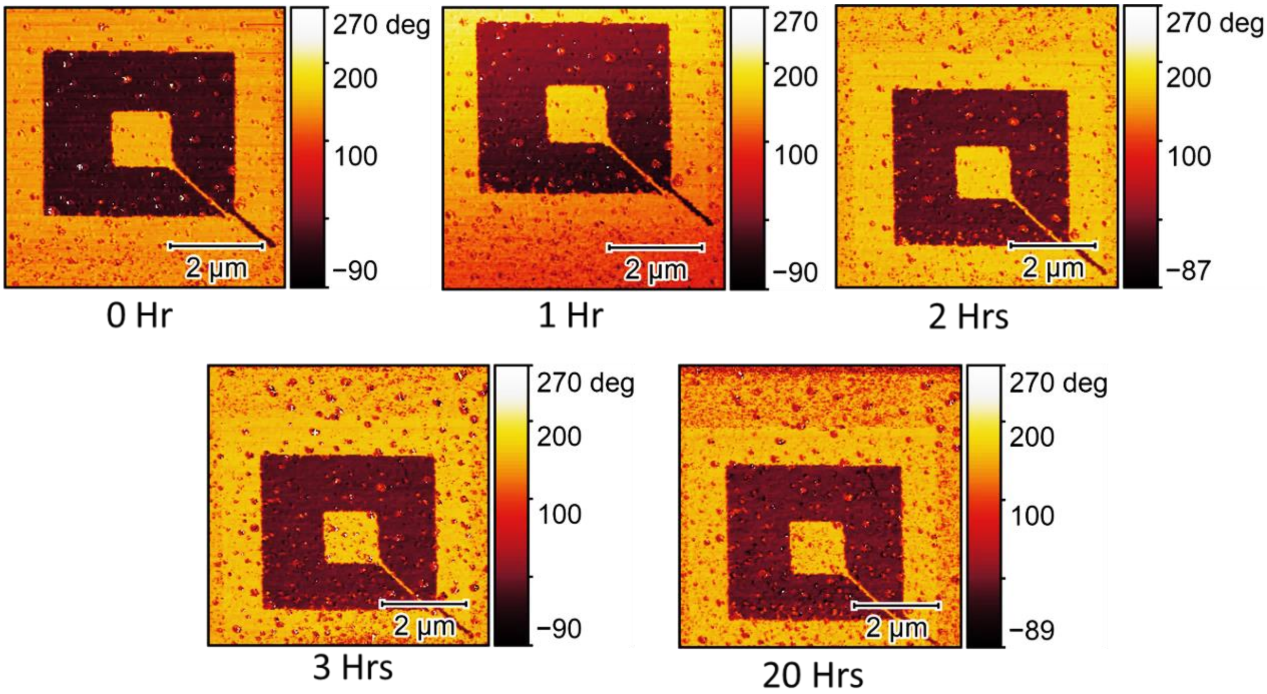


**Supplementary Figure 9|** PFM measured for at least 24 hours after writing the box-in-box at ±5V in sample BPBO/BLFO. The images were recorded on a sample deposited at 450°C. The PFM tip location appears to be shifted slightly but the polarization phase contrast does not change.

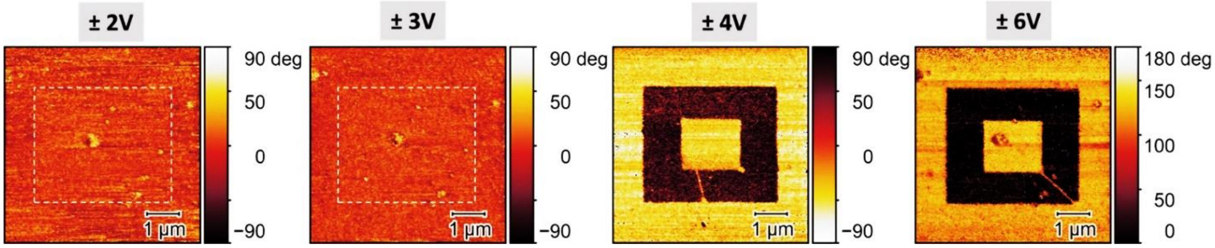


**Supplementary Figure 10|** Bias-voltage-dependent PFM phase images of BPBO/BLFO.

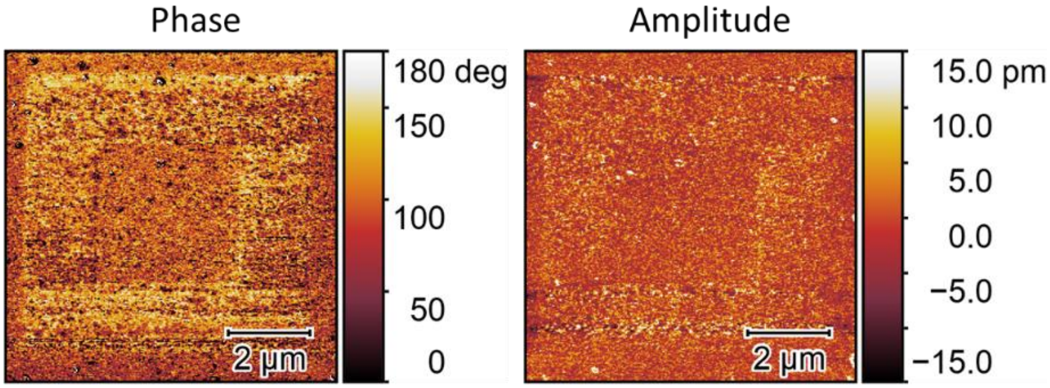


**Supplementary Figure 11|** PFM images of SRO/BLFO deposited at 450°C. The piezoelectric force microscopy was recorded in the same way as in Figure 3 (main text) box-in-box at ±6V. We do not see any phase reversal in the polarization contrast, which is expected as no ferroelectricity hysteresis loops were visible (Figure 4a) at 450°C in SRO/BLFO/SRO sample.

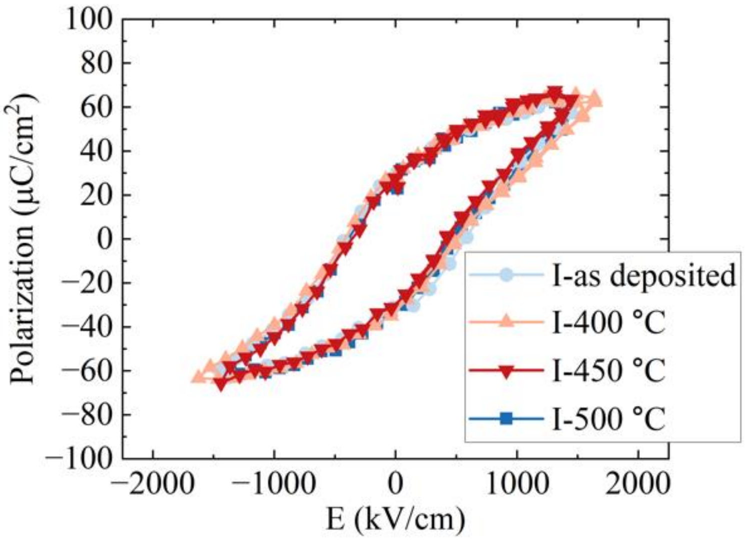


**Supplementary Figure 12|** Ferroelectric hysteresis recorded in sample BPBO/BLFO/BPBO prepared in different temperatures post-annealing. The four BPBO/BLFO/BPBO samples were grown in the same deposition run to understand any impact of the post-annealing on the ferroelectric properties. The samples were post-annealed up to 500°C in steps of 50°C. The ferroelectric properties don't show appreciable change in the hysteresis after annealing whereas the fatigue data (main text Figure 5) shows an improvement which in indicative of improved defect centers if we do post-annealing after the deposition.

## Supplementary Note 2. Work function and band diagram in BPBO and SRO-based heterostructures.

Here we compare the SRO/BLFO/SRO and BPBO/BLFO/BPBO heterostructures energy band based on the material's work functions. First, we measured the carrier concentration type (n-or p-type) to draw the band schematic. X-ray diffraction pattern (Supplementary Figure 13a) and longitudinal resistivity (Supplementary Figure 13c) confirmed the growth of SRO thin film on STO. From the slope of the $R_{xy}$ vs. the out-of-plane magnetic field ($\mu_0 H$) (Supplementary Figure 13d), the carrier concentration is found to be ~$1.2\times10^{23}$ $cm^{-3}$ at room temperature. A negative slope is indicative of *n*-type carriers in the SRO.

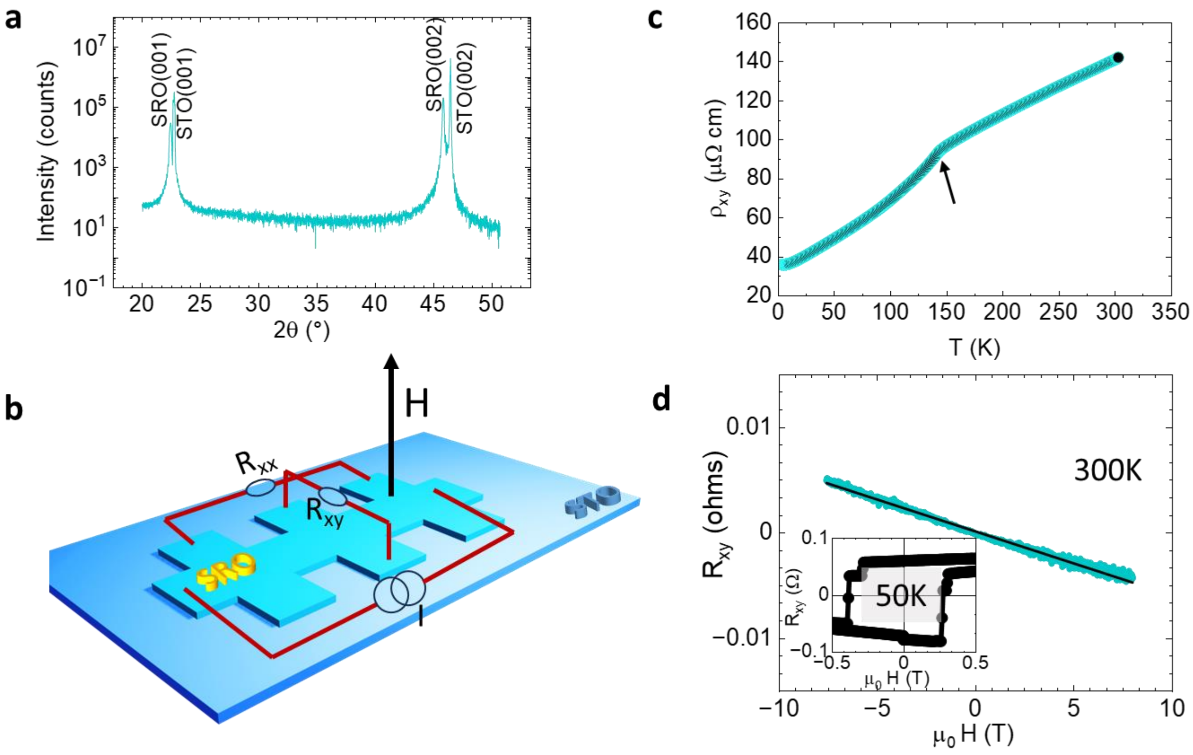


**Supplementary Figure 13| N-type metal SRO. a** X-ray diffraction pattern of SRO (75nm)/STO. **b** Patterned Hall bar (6μm wide strip) of SRO (75 nm) along with the measurement circuit. **c** Longitudinal resistivity as a function of temperature; arrow indicates the ferromagnetic transition in SRO. **d** Measured transverse resistance ($R_{xy}$) as a function of out-of-plane magnetic field (±8 T) at constant 1mA dc at 300K; inset shows the perpendicular magnetic anisotropy hysteresis measured at 50K. Line is fit to the data to calculate the carrier concentration at 300K.

Invigorated with the carrier type of SRO, it is reported to be metal with a work function of 5.2eV[5] and BLFO is insulating with a work function of 4.7eV [5]. $BaBiO_3$ is *p*-type conductor with work function of 4.2eV[6]. In case of SRO/BLFO/SRO, the Fermi level of SRO lies below the BLFO forming a Schottky barrier with a positive slope where the electrons require energy to excite into the BLFO and form the charge on the interface to pole the LBFO dielectric and opposite charge build-up on the other side of the BLFO/electrode interface. In case of BPBO/BLFO/BPBO, the BPBO is *p*-type conductor where the Fermi level of BLFO lies at lower energy than that of BPBO. In this case, the electronic losses their energy during hopping.

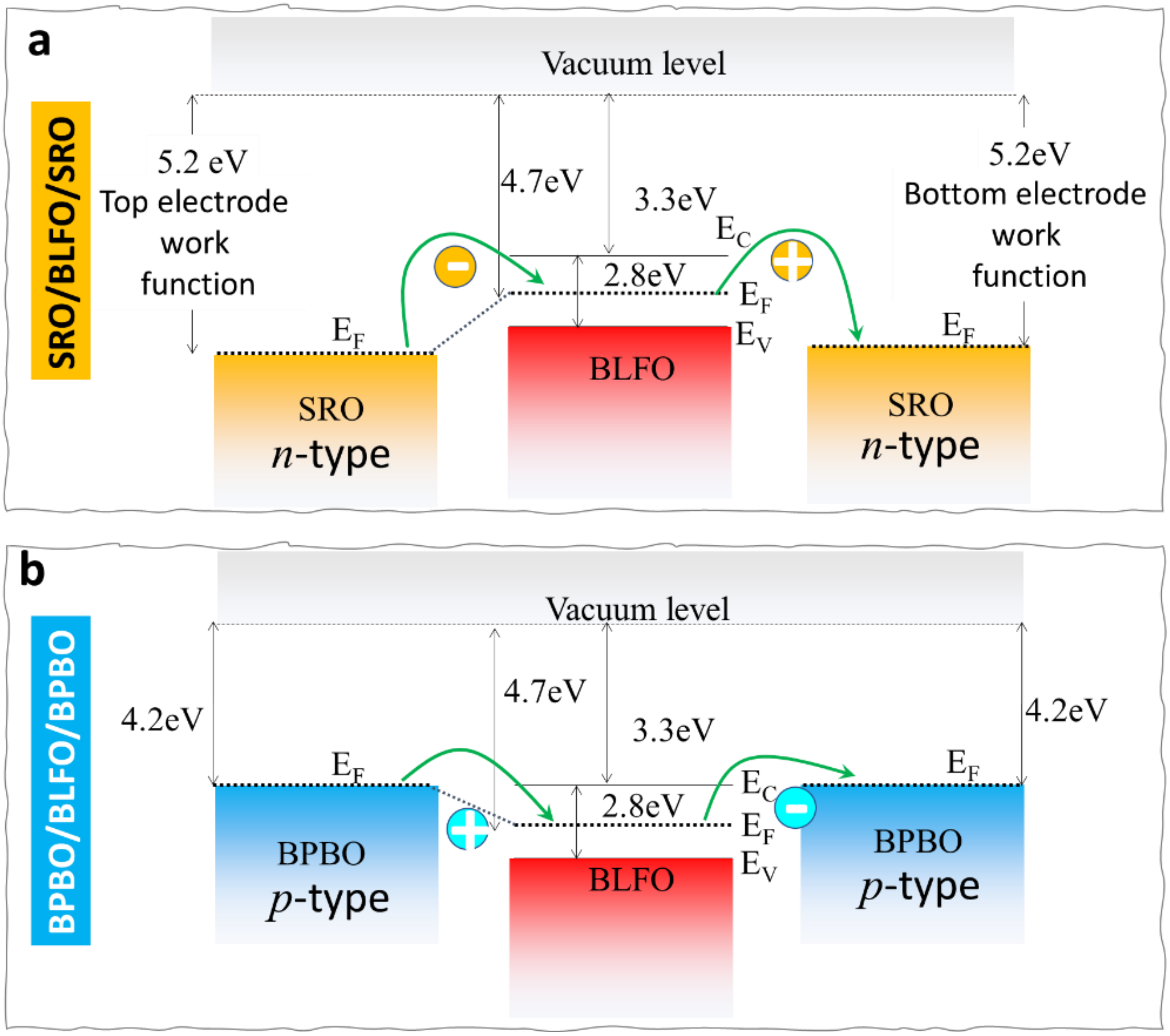


**Supplementary Figure 14|** Energy band schematics for **a** SRO/BLFO/SRO and **b** BPBO/BLFO/BPBO.

## References:


1. Kubel, B. F. & Schmid, H. Structure of ferroelectric and ferroelastic monodomain crystal of the perovskite BiFeO3. *Acta Cryst.* **B46**, 698–702 (1990).
2. Huang, Y. *et al.* Manipulating magnetoelectric energy landscape in multiferroics. *Nat Commun* **11**, 2836 (2020).
3. Farokhipoor, S. & Noheda, B. Conduction through 71°domain walls in BiFeO3 thin films. *Phys Rev Lett* **107**, 127601 (2011).
4. Li, Z. *et al.* Reversible manipulation of the magnetic state in SrRuO3 through electric-field controlled proton evolution. *Nature Communications 2020 11:1* **11**, 184 (2020).
5. Wang, C. *et al.* Switchable diode effect and ferroelectric resistive switching in epitaxial BiFeO3 thin films. *Appl Phys Lett* **98**, 192901 (2011).
6. Chouhan, A. S., Athresh, E., Ranjan, R., Raghavan, S. & Avasthi, S. BaBiO3: A potential absorber for all-oxide photovoltaics. *Mater Lett* **210**, 218–222 (2018).
