# Supplementary material for: Low-temperature grapho-epitaxial La-substituted BiFeO3 on metallic perovskite
Source: Nat Commun. 2024 Jan 11;15:479. doi: 10.1038/s41467-024-44728-y (PMC10784590; doi:10.1038/s41467-024-44728-y)
Supplement: Supplementary file 3 — Reporting Summary [file 41467_2024_44728_MOESM3_ESM.pdf]

nature research

Corresponding author(s): Sajid Husain

Double-blind peer review submissions: write DBPR and your manuscript number here instead of author names.

☐ Initial submission ☐ Revised version ☑ Final submission

Lasing Reporting Summary

Nature Research wishes to improve the reproducibility of the work that we publish. This form is intended for publication with all accepted papers reporting claims of lasing and provides structure for consistency and transparency in reporting. Some list items might not apply to an individual manuscript, but all fields must be completed for clarity.

For further information on Nature Research policies, including our data availability policy, see Authors & Referees.

Experimental design

Please check: are the following details reported in the manuscript?

1. Threshold

Plots of device output power versus pump power over a wide range of values indicating a clear threshold
☐ Yes — State where this information can be found in the text.
☑ No — Explain why this information is not reported/not relevant.

2. Linewidth narrowing

Plots of spectral power density for the emission at pump powers below, around, and above the lasing threshold, indicating a clear linewidth narrowing at threshold
☐ Yes — State where this information can be found in the text.
☑ No — Explain why this information is not reported/not relevant.

Resolution of the spectrometer used to make spectral measurements
☐ Yes — State where this information can be found in the text.
☑ No — Explain why this information is not reported/not relevant.

3. Coherent emission

Measurements of the coherence and/or polarization of the emission
☐ Yes — State where this information can be found in the text.
☑ No — Explain why this information is not reported/not relevant.

4. Beam spatial profile

Image and/or measurement of the spatial shape and profile of the emission, showing a well-defined beam above threshold
☐ Yes — State where this information can be found in the text.
☑ No — Explain why this information is not reported/not relevant.

5. Operating conditions

Description of the laser and pumping conditions
Continuous-wave, pulsed, temperature of operation
☐ Yes — State where this information can be found in the text.
☑ No — Explain why this information is not reported/not relevant.

Threshold values provided as density values (e.g. W cm^{-2} or J cm^{-2}) taking into account the area of the device
☐ Yes — State where this information can be found in the text.
☑ No — Explain why this information is not reported/not relevant.

6. Alternative explanations

Reasoning as to why alternative explanations have been ruled out as responsible for the emission characteristics
e.g. amplified spontaneous, directional scattering; modification of fluorescence spectrum by the cavity
☐ Yes — State where this information can be found in the text.
☑ No — Explain why this information is not reported/not relevant.

7. Theoretical analysis

Theoretical analysis that ensures that the experimental values measured are realistic and reasonable
e.g. laser threshold, linewidth, cavity gain-loss, efficiency
☐ Yes — State where this information can be found in the text.
☑ No — Explain why this information is not reported/not relevant.

8. Statistics

Number of devices fabricated and tested
☐ Yes — State where this information can be found in the text.
☑ No — Explain why this information is not reported/not relevant.

Statistical analysis of the device performance and lifetime (time to failure)
☐ Yes — State where this information can be found in the text.
☑ No — Explain why this information is not reported/not relevant.

FOR REFERENCE ONLY

Further reading

We also suggest that authors read the following literature, which describes the important principles and signatures of laser emission and discusses some of the common mistakes that can occur during laser characterization.

1. Samuel I.D.W., Namdas, E.B. & Turnbull, G.A. How to recognize lasing. *Nat. Photon.* **3**, 546-549 (2009).
2. Siegmann, A.E. *Lasers*. (University Science Books, 1990)
3. Svelto, O. *Principles of Lasers*. 5th edn. (Springer 2010)
4. Blood, P. *Quantum Confined Laser Devices: Optical Gain and Recombination in Semiconductors*. (Oxford Univ. Press, 2015)
5. Koxlov, V.G. *et al*. Laser action in organic semiconductor waveguide and double-heterostructure devices. *Nature* **389**, 362-364 (1997).

FOR REFERENCE ONLY

This checklist template is licensed under a Creative Commons Attribution 4.0 International License, which permits use, sharing, adaptation, distribution and reproduction in any medium or format, as long as you give appropriate credit to the original author(s) and the source, provide a link to the Creative Commons license, and indicate if changes were made. The images or other third party material in this article are included in the article's Creative Commons license, unless indicated otherwise in a credit line to the material. If material is not included in the article's Creative Commons license and your intended use is not permitted by statutory regulation or exceeds the permitted use, you will need to obtain permission directly from the copyright holder. To view a copy of this license, visit http://creativecommons.org/licenses/by/4.0/
